# The London Hospital's Nurses

**Published:** 1918-10-12

**Authors:** 


*To the Editor of* The Hospital.

Sir,—Your readers must be wondering why there has not been a greater protest from the London Hospital nursing staff against Dr. Chapple's unwarrantable attack on them and their training-school. It is not from lack of loyalty to our hospital, for we all feel strongly enough about it, but most of us feel that letters of this sort are best left unanswered.

They are not written from a desire to *help* anyone, but from personal animosity; and amongst all clear-sighted people Dr. Chapple must be doing more harm to himself than to us.

He complains about our training, but surely the doctors for whom we work and our patients are better judges of that than he can be; and as to our agreement with the hospital, we fully understand the conditions under which we enter for our training and the arrangements made afterwards, and we object to his interference and do not need his help.

If we desired any alteration we should go to our matron or chairman, who have done more than anyone since the days of Florence Nightingale to improve the conditions under which nurses work, and certainly not to Dr. Chapple, who is in no way connected with our hospital nor in the least interested in our welfare.

Our committee were the first to raise the salaries of their nursing staff to meet the present war conditions, and we know they will continue to do so as it becomes possible.

The large majority of us stay on for many years after our term of agreement is ended because we want to. We are happy here and exceedingly well cared for, and we know there are few places where we could carry out our work under such excellent conditions.—I am, Sir, yours truly, A Present Member of the Nursing Staff, London Hospital.

October 7, 1918.

